# Graphene Supported NiFe-LDH and PbO_2_ Catalysts Prepared by Plasma Process for Oxygen Evolution Reaction

**DOI:** 10.3390/ma18010121

**Published:** 2024-12-31

**Authors:** Tingting Yang, Zheng Zhang, Fei Tan, Huayu Liu, Xingyu Li, Hongqi Wang, Qing Yang

**Affiliations:** 1 State Grid Chongqing Electric Power Company Material Branch, Chongqing 401120, China; 2 Chongqing Jie Chuang Electric Power Technology Company Ltd., Chongqing 400031, China; 3 State Key Laboratory of Power Transmission Equipment Technology, School of Electrical Engineering, Chongqing University, Chongqing 400044, China

**Keywords:** plasma applications, graphene, vacancies, electrocatalysis, OER

## Abstract

The development of efficient catalysts for water electrolysis is crucial for advancing the low-carbon transition and addressing the energy crisis. This work involves the fabrication of graphene-based catalysts for the oxygen evolution reaction (OER) by integrating NiFe-LDH and PbO_2_ onto graphene using plasma treatment. The plasma process takes only 30 min. Graphene’s two-dimensional structure increases the available reaction surface area and improves surface electron transport. Plasma treatment further improves catalyst performance by facilitating nanoparticle attachment and creating carbon defects and sulfur vacancies. Density functional theory (DFT) calculations at the PBE provide valuable insights into the role of vacancies in enhancing catalyst performance for OER. The catalyst’s conductivity and electronic structure are greatly impacted by vacancies. While modifications to the electronic structure increase the kinetics of charge transfer, the vacancy structure can produce more active sites and improve the adsorption and reactivity of OER intermediates. This optimization of intermediate adsorption and electronic properties leads to increased overall OER activity. The catalyst NiFe-PbO_2_/S/rGO-45, synthesized through plasma treatment, demonstrated an overpotential of 230 mV at 50 mA·cm^−2^ and a Tafel slope of 44.26 mV dec^−1^, exhibiting rapid reaction kinetics and surpassing the OER activity of commercial IrO_2_. With its excellent performance, the prepared catalyst has broad prospects in commercial applications such as water electrolysis and air batteries.

## 1. Introduction

To meet the demands of energy structure adjustment and ensure the safe and stable operation of power grids, the integration of renewable energy sources is becoming increasingly imperative. A potential energy source, hydrogen is capable of being created from a variety of non-renewable and renewable resources. Hydrogen is distinguished for its high calorific value and environmentally benign combustion properties, as it only produces water as a byproduct when used in fuel cells or combustion engines. These characteristics make hydrogen an attractive option [1,2,3]. Water electrolysis, particularly when powered by renewable energy, is a key technology for achieving carbon peak and carbon neutrality goals. Nonetheless, the OER occurring at the anode during water electrolysis poses a considerable challenge. This process involves a complex four-electron transfer, which is kinetically slow, requires a high overpotential, and results in low reaction efficiency—factors that hinder the widespread adoption of water electrolysis technology [4,5]. To tackle these challenges, effective OER catalysts that could enhance performance and reduce the reaction energy barrier require being developed.

Currently, most OER catalysts rely on precious metals or their compounds. Despite the high catalytic activity, high cost and limited availability hinder large-scale applications [6,7]. In contrast, transition metals are garnering significant interest because of their plentiful reserves, affordability, and effective catalytic properties. Nickel-iron bimetallic catalysts, in particular, have gained widespread interest for their high catalytic activity and relatively lower cost [8,9]. The combined influence of nickel and iron lowers the energy barrier of OER, thereby enhancing catalytic efficiency. However, NiFe bimetallic catalysts still face several practical challenges, such as poor stability of active sites, insufficient electrical conductivity, and reduced catalytic activity caused by the formation of surface oxides. To overcome these issues, recent research has suggested that incorporating PbO_2_ and graphene into the catalyst could significantly improve its performance. PbO_2_ is utilized because of its high conductivity, which compensates for the poor conductivity of transition metals. At the same time, since lead dioxide can be obtained from waste lead-acid batteries, the use of lead dioxide can promote the recycling of lead-acid batteries [10]. Graphene is known for its excellent redox properties and high electrical conductivity. The combination of PbO_2_ and graphene helps enhance charge transfer efficiency, stabilize the catalyst surface, and alleviate the decrease of active sites, consequently improving the durability and catalytic efficacy [11,12]. Furthermore, the development of vacancy structures in graphene and the associated compounds during synthesis has the potential to increase the reaction surface area, thereby improving catalytic efficiency. For example, Rocha et al. fabricated a multi-vacancy, doped low-concentration nickel-reduced graphene oxide 3D aerogel through freeze-casting, achieving an overpotential of 480 mV [13]. Fei et al. created a porous core-shell nanoreactor that encapsulates NiFe within graphene layers. This design exhibited remarkable stability, operating for 2000 h under alkaline seawater conditions [14]. These studies highlight the potential of PbO_2_ and graphene, along with vacancy structures, to address the limitations of transition metals in OER catalysts. However, the underlying mechanisms of these improvements still require further investigation.

Herein, this work examines the impact of vacancy structures on the performance of graphene-supported transition metals and PbO_2_, employing RF plasma treatment to integrate NiFe-LDH and PbO_2_ onto graphene. The analysis focuses on the morphological structure and electrochemical performance, aiming to uncover their surface catalytic activity and offer insights into the underlying microscopic reaction mechanisms.

## 2. Materials and Methods

### 2.1. Materials

The materials utilized include commercial graphene oxide powder (GO, purity 99.5%, thickness 6–8 nm, width 25 µm) sourced from Ni(NO_3_)_2_·6H_2_O, FeSO_4_·7H_2_O, thiourea, and PbO_2_, all of analytical grade, also obtained from Macklin; and anhydrous ethanol, which is of analytical grade, acquired from Chengdu Kelong.

### 2.2. Preparation of NiFe-PbO_2_/S/rGO-X

A precursor solution was prepared by adding the following materials to 30 mL of deionized water: 30 mg of graphene oxide, 145.395 mg of nickel nitrate hexahydrate, 139.025 mg of iron (II) sulfate heptahydrate, 111.6 mg of lead dioxide, and 228.36 mg of thiourea. To ensure complete dissolution, the solution was sonicated for half an hour. To get the precursor powder sample, the solution was then freeze-dried for 24 h at −75 °C.

**Figure 1:**
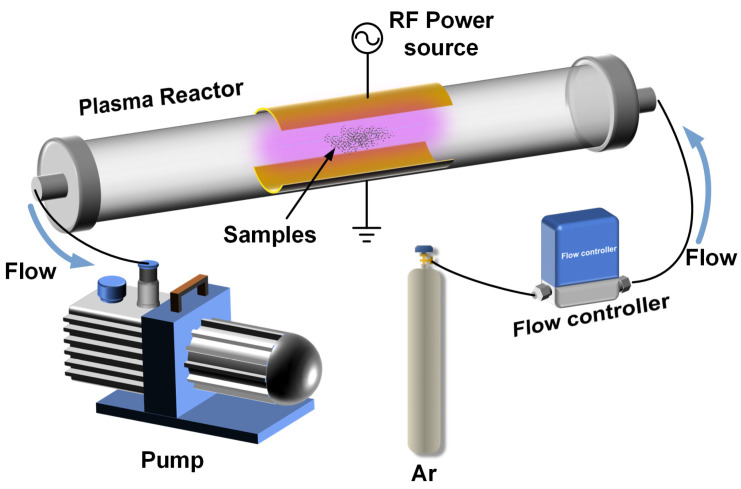
RF plasma experimental platform.

**Figure 2:**
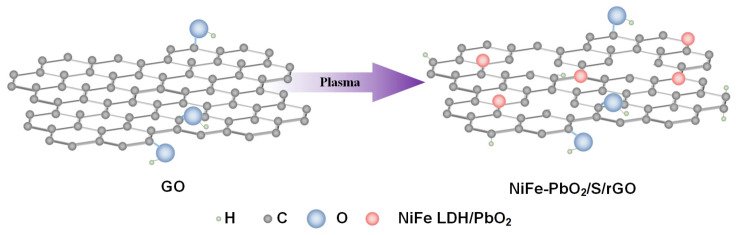
Preparation process of NiFe-PbO_2_/S/rGO-X catalyst.

An RF plasma-assisted treatment experimental platform was established, as shown in Figure 1. The system consists of a radio frequency (RF) power generator (Beijing Jinmao Technology Co., Ltd., Beijing, China), a matching network, and a vacuum system, which work together to generate plasma for the treatment process. A copper plate wrapped around a quartz tube serves as the electrode, which is connected to the RF power supply. Argon gas (Ar, purity 99.999%) is introduced into the reaction chamber to generate the plasma. The treatment process begins by placing the precursor inside the chamber, followed by the introduction of argon gas under vacuum conditions at a flow rate of 50 sccm. The plasma is then produced utilizing an RF power supply functioning at a frequency of 13.56 MHz and a power output of 80 W. The precursor undergoes plasma treatment for varying durations, which allows the control of vacancy structure development. NiFe-PbO_2_/S/rGO-X catalyst samples are obtained, where “X” refers to the treatment time of plasma (X = 15, 30, 45, 60, min), with each sample corresponding to a specific degree of vacancy structure formation (Figure 2).

### 2.3. Material Characterization

The catalyst was characterized using a variety of analytical techniques to study its structure and composition. X-ray diffraction (XRD) research was conducted utilizing a D8 Advance (Bruker, Bremen, Germany) at a scanning velocity of 0.2°/s to investigate the crystalline structure. Raman spectra were acquired using a 532 nm laser Raman microscope equipment (Renishaw, London, UK) to examine the crystal phase. Scanning electron microscopy (SEM) pictures were obtained with a S-4800 (Hitachi, Tokyo, Japan) to examine the surface morphology of the catalyst. X-ray photoelectron spectroscopy (XPS) was performed using an ESC-Lab-250Xi (Thermo Fisher Scientific Inc., Waltham, MA, USA) with monochromatic Al-Kα radiation to analyze the chemical states of the elements. Electron paramagnetic resonance (EPR) tests utilizing the A30010/12 system (Bruker, Bremen, Germany) were conducted to investigate the structure of the detector. The elemental concentrations were measured using inductively coupled plasma mass spectrometry (ICP-MS, ICP OES 7200, Thermo Fisher Scientific Inc., Waltham, MA, USA). The emission spectra of the materials were acquired utilizing an MX2500+ spectrometer (Ocean Optics, Orlando, FL, USA), covering a wavelength range of 200 nm to 1100 nm.

### 2.4. Preparation of the Ink of Working Electrode

Prepare a solution by dissolving 10 mg of the catalyst sample in a combination of 400 µL anhydrous ethanol and 40 µL 0.5% Nafion solution. Subject the mixture to sonication for a duration of 30 min, then permit it to stand for subsequent utilization. To facilitate comparison, dissolve 10 mg of IrO_2_ (sourced from Shanghai Macklin, analytical grade) in the identical solvent mixture—400 µL anhydrous ethanol and 40 µL 0.5% Nafion solution. Sonicate for 30 min and set aside for later use.

### 2.5. Electrochemical Characterization

Electrochemical performance evaluation was conducted with a three-electrode configuration on a CHI 760 (Shanghai Chenhua Instrument Co. Ltd, Shanghai, China). The working electrode was fabricated by depositing 100 µL catalyst slurry onto a 1 × 1 cm^2^ nickel foam substrate. A platinum electrode functioned as the counter electrode, whereas a silver/silver chloride electrode was employed as the reference electrode. All experiments were performed in an O_2_-saturated 1 M KOH. The calibration equation employed for potential measurements was *E*_RHE_ = *E*_Ag/AgCl_ + 0.0591 × pH + 0.197. Cyclic voltammetry (CV) measurements were conducted at a scan rate of 10 mV s^−1^ to evaluate the electrochemical properties of the catalyst. Electrochemical impedance spectroscopy (EIS) was conducted over a frequency range of 10^−2^ Hz to 10^5^ Hz to examine the correlation between impedance and OER at different applied potentials. The current density was assessed for a duration of 50 h by amperometric i–t curves at a constant voltage of 1.52 V to evaluate long-term stability. The catalyst’s OER activity was further investigated by linear sweep voltammetry (LSV) at a scan rate of 5 mV s^−1^, within a potential range of 1–2 V vs. RHE. The double-layer capacitance (*C*_dl_) method was employed to investigate the catalyst’s electrochemical active surface area (ECSA) and improve catalytic performance. CV measurements were performed at many scan speeds inside an electrochemical window devoid of redox processes, facilitating the determination of *C*_dl_.

### 2.6. Computational Details

Density functional theory (DFT) was performed to determine the energy and structure of each OER intermediate in two heterostructure models: NiFe-PbO_2_/S/V_G_ and V_G_ and Graphene. In these models, plasma deposition is tackled by the DFT-backed synthetic growth concept; NiFe and PbO_2_ were supported on a (4 × 4) supercell of graphene with vacancy defects. An optimized model of graphene, an optimized model of vacancy-defect graphene, was established for comparison. The layered two-dimensional structure of graphene was established based on the reported research [15,16]. The substrates were formed by NiFe (003) and PbO_2_ (111) crystal planes. The computations were performed utilizing CASTEP with the Perdew-Burke-Ernzerhof (PBE) exchange-correlation functional under the generalized gradient approximation (GGA). The U_eff_ values for nickel and iron were established at 6.6 eV and 3.5 eV, respectively. Additionally, we used the Nose-Hoover thermostats at 300 K and 600 K, respectively, to perform ab initio molecular dynamics (AIMD) simulations in the NVT canonical ensembles. For the AIMD simulations, the time step was 2.0 fs and the overall time was 10 ps.

## 3. Results and Discussion

**Figure 3:**
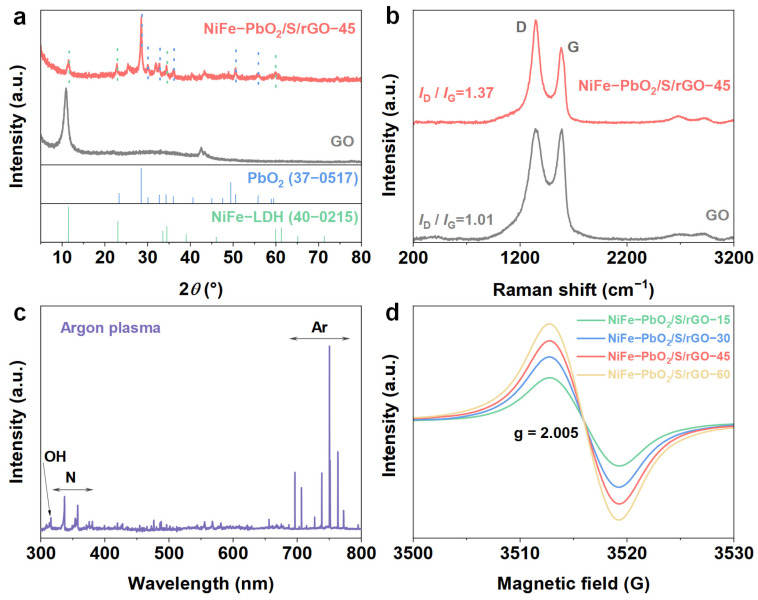
(**a**) XRD patterns of NiFe-PbO_2_/S/rGO-45, (**b**) Raman spectra of NiFe-PbO_2_/S/rGO-45 and GO, (**c**) Optical emission spectrum of Argon plasma, (**d**) EPR spectrum of NiFe-PbO_2_/S/rGO-X.

XRD and Raman were employed to examine the structural properties of NiFe-PbO_2_/S/rGO-X. As shown in Figure 3a, XRD analysis of NiFe-PbO_2_/S/rGO-45 reveals several diffraction peaks corresponding to NiFe LDH (JCPDS No. 40-0215) at 11.4°, 22.9°, 34.4°, and 59.8°, which correspond to the (003), (006), (012), and (110) crystal planes [17]. Additionally, peaks at 28.5°, 30°, 32.7°, 36°, 50.6°, and 55.8° correspond to the (111), (020), (002), (200), (221), and (113) crystal planes of PbO_2_ (JCPDS No. 37-0517), confirming the loading of NiFe-LDH and PbO_2_ onto the graphene surface.

Raman spectroscopy further examined the defective structure of the graphene. The D peak at 1350 cm^−1^, which indicates the presence of defects, and the G peak at 1580 cm^−1^, which represents the graphitic structure, demonstrate that plasma treatment induces defects in the graphene [18,19]. The intensity ratio of the D peak to the G peak (*I*_D_/*I*_G_) for NiFe-PbO_2_/S/rGO-45 is 1.37. This value markedly surpasses the ratio of 1.01 for graphene oxide (GO), suggesting that the Ar plasma treatment effectively etches the graphene, creating a large number of defects while preserving its carbon framework. These defects enhance ion adsorption, facilitate carrier migration, and provide sites for the accommodation of NiFe LDH and PbO_2_ nanoparticles. Additionally, the plasma treatment modifies the graphene’s electronic band structure, promoting hole-type carriers and increasing the band gap, which facilitates sulfur doping and the formation of catalytic active sites [20]. Furthermore, carbon vacancies in graphene-based materials can also enhance their catalytic activity towards the OER. Highly localized states close to the Fermi level are introduced by carbon vacancies, and these states can operate as active sites for the adsorption and activation of OER intermediates [21]. The highly localized states introduced by carbon vacancies facilitate the adsorption and activation of OER intermediates, reducing the overpotential required for the reaction, and act as anchoring sites for metal nanoparticles, promoting the dispersion and stability of the catalytically active phase, further enhancing OER activity [22].

OES is an efficient technique for analyzing the active species and their physicochemical characteristics (Figure 3c). It offers significant insights into the origin of active particles in the sample, facilitating the comprehension of the effects of plasma on the NiFe-PbO_2_/S/rGO-X lattice [23]. Plasma treatment with argon dramatically modifies the properties of the lattice by introducing high-energy particles and reactive functional groups (Ar, -OH, -N). The species in question are capable of initiating redox reactions, which leads to the formation of sulfur vacancies as catalytic active sites, crucial for improving the efficacy of the catalyst [24].

The catalyst’s surface is struck by the charged particles in the plasma, which promotes atomic sputtering and increases the number of vacancy defects. The high temperature of plasma promotes the formation of intermediate products such as H_2_S and NH_3_ from thiourea, while plasma-induced ionization and excitation of gas molecules facilitate S and N doping into the graphene structure. Furthermore, free electrons, ions, and atoms inside the plasma engage with the GO surface, adsorbing negatively charged oxygen functional groups that subsequently undergo reduction, hence augmenting the material’s conductivity. The plasma etching effect creates nanopores on the rGO surface, providing low-resistance channels for O_2_ and increasing the surface area for metal oxide nanoparticle support.

Consequently, plasma treatment interferes with the C-S and S-S bonds in S-doped rGO, resulting in the creation of vacancies that modify the electronic structure. This technique facilitates concurrent material loading and etching, producing a synergistic action that boosts the OER catalytic efficacy. Post-plasma treatment, the number of active sites on the catalyst surface escalates, augmenting the reactive area and enhancing catalytic activity [25,26].

EPR measurements were conducted to further validate the existence of vacancy structures in the NiFe-PbO_2_/S/rGO-X composite (Figure 3d). An identifiable S vacancy signal was at g = 2.005 [27]. S vacancies cause unsaturated sites to develop, which operate as active sites for the adsorption and activation of OER intermediates because they have higher electron densities and better charge transfer capacities [28]. This leads to a reduction in the overpotential required for OER, enhancing the catalytic activity [29]. Moreover, the intensity of the EPR signal increased with plasma etching duration, signifying a progressive rise in the number of vacancies. This indicates that modifying the plasma treatment duration allows for effective control over vacancy defects, which in turn promotes better electron transfer and enhanced catalytic activity.

**Figure 4:**
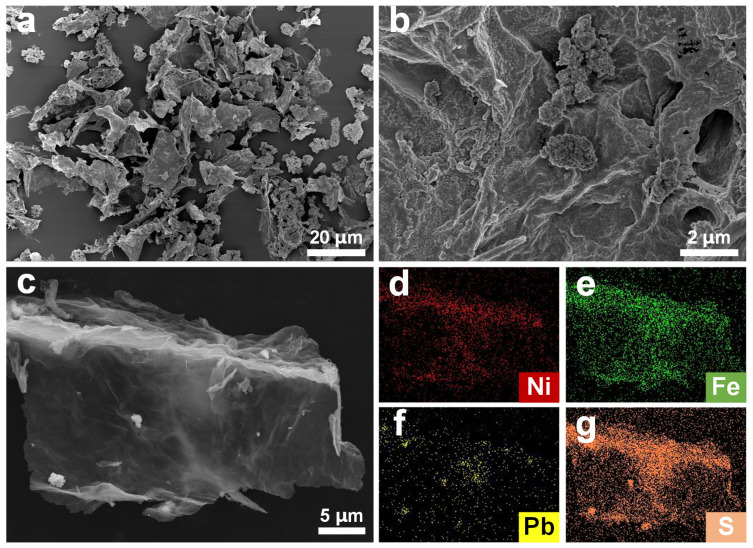
(**a**) SEM images of NiFe-PbO_2_/S/rGO-45, (**b**) SEM images of NiFe-PbO_2_/S/rGO-45, SEM mapping area of (**c**) NiFe-PbO_2_/S/rGO-45, and the elemental distribution of (**d**) Ni, (**e**) Fe, (**f**) Pb, (**g**) S.

To investigate the effect of plasma treatment on the microstructure of NiFe-PbO_2_/S/rGO-X, SEM was used for characterization. As shown in Figure 4a,b, plasma treatment exfoliates GO into reduced graphene oxide (rGO) nanosheets. This reduction is driven by free active radicals from the Ar plasma, which interact with the GO surface. GO consists of both *sp*^2^ and *sp*^3^ domains, with *sp*^2^ representing the unoxidized regions and *sp*^3^ representing the oxidized regions. The high-energy particles of plasma erode GO, cleaving carbon-oxygen bonds and eliminating oxygen atoms. Furthermore, free electrons, ionized argon molecules, and atoms from the plasma engage with the graphene oxide surface, resulting in the desorption of oxygen functional groups and the diminution of *sp*^3^ domains. This results in the reduction of GO, facilitated by the plasma treatment, which plays a crucial role in removing oxygenated functional groups.

EDS mapping further indicates that Ni, Fe, Pb, and S are homogeneously distributed across the rGO surface (Figure 4c–g and Figure S1). NiFe LDH and PbO_2_ are loaded onto rGO as nanoparticles (NiFe-PbO_2_/S/rGO-45), which significantly enhances the material’s electrocatalytic performance. The nanoparticle diameter is approximately 29 nm (Figure S2), and their distribution on the rGO surface facilitates water molecule adsorption and accelerates electron transfer, further improving the activity.

**Figure 5:**
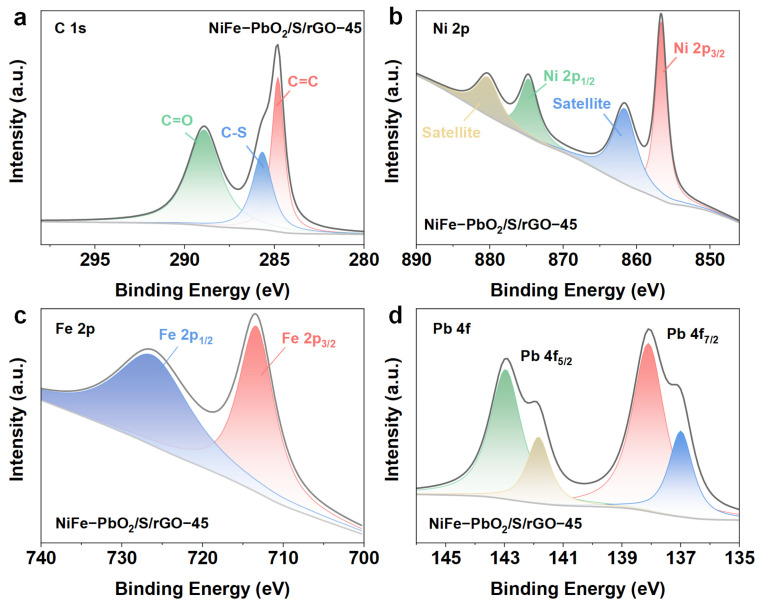
XPS spectrum of NiFe-PbO_2_/S/rGO-45 (**a**) Ni 2p, (**b**) Fe 2p, (**c**) Pb 4f, (**d**) S 2p.

XPS was used to further examine the impact of plasma treatment on the chemical states of NiFe-PbO_2_/S/rGO-X. Figure S3 illustrates that the detection of N 1s and S 2p peaks in the XPS full spectrum of NiFe-PbO_2_/S/rGO-45 signifies that high-energy plasma activates anionic precursors, producing active free radicals that enhance element doping. Plasma-induced groups specifically degrade thiourea, yielding the intermediate product S^2-^. The sulfur species are then stimulated or ionized in the plasma, resulting in the integration of free sulfur atoms into the graphene structure. The C 1s fine spectra (Figure 5a) display 284.8 eV, 285.7 eV, and 288.9 eV, which are attributed to C-C, C-S, and C=O bonds, respectively. In comparison to the C 1s fine spectrum of GO of comparable purity documented in the literature, the strength of the C=O peak in NiFe-PbO_2_/S/rGO-45 is markedly diminished, thereby validating the effective reduction of GO to rGO through the elimination of oxygen species [30,31]. The peak fitting of the Ni 2p fine spectrum (Figure 5b) identifies four unique chemical states of Ni. 856.7 eV and 874.5 eV correspond to the Ni 2p_3/2_ and Ni 2p_1/2_ orbitals linked to Ni^3+^ ions. Ni^3+^ ions can function as p-type dopants by introducing holes or as n-type dopants by introducing additional electrons. 861.7 eV and 880.4 eV are satellite peaks [32,33]. The Fe 2p spectrum (Figure 5c) exhibits 713.1 eV and 726.3 eV, which correspond to Fe^3+^ ions, signifying the oxidation of Fe^2+^ to Fe^3+^ during plasma treatment [34]. The high-resolution Pb 4f spectrum (Figure 5d) has prominent peaks at 137 eV and 141.8 eV, indicative of Pb^4+^, and at 138.1 eV and 143 eV, representative of Pb^2+^. This suggests that the Pb ions in NiFe-PbO_2_/S/rGO-45 exist in a mixed valence state of Pb^2+^ and Pb^4+^. The presence of Pb vacancies adjacent to Pb ions provides additional electrons to the O ions, which have detached from the Pb, thereby achieving charge balance and enhancing the catalytic properties [35].

**Figure 6:**
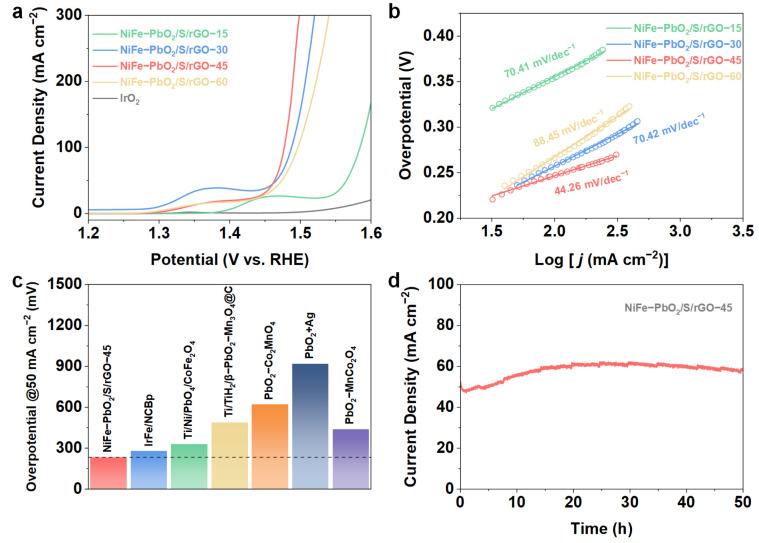
OER performances of NiFe-PbO_2_/S/rGO-45. (**a**) LSV curve, (**b**) Tafel slope, (**c**) Comparison of η_50_. (**d**) Amperometric i–t curve.

The OER activity of NiFe-PbO_2_/S/rGO-X was assessed utilizing a three-electrode setup in a 1 mol·L^−1^ KOH, with commercial IrO_2_ examined under identical circumstances for comparative analysis. As shown in Figure 6a, the LSV polarization curves reveal that NiFe-PbO_2_/S/rGO-45 exhibits the lowest overpotential, with an η_50_ of 230 mV, significantly lower than the commercial catalyst IrO_2_ (η_50_ for 50 mA·cm^−2^ overpotential), and superior to similar OER catalysts reported in recent literature (Figure 6c) [36,37,38,39,40,41]. However, as the plasma treatment time increased, the overpotential did not continue to decrease, likely due to the excessive formation of vacancy defects, which could lead to structural instability of the catalyst. In Figure 6b, the Tafel slope of NiFe-PbO_2_/S/rGO-45 (44.26 mV·dec^−1^) is the lowest, indicating fast reaction kinetics. This is further supported by the EIS results (Figure S4), which show that NiFe-PbO_2_/S/rGO-45 has the lowest charge transfer impedance. The enhanced conductivity and electron transfer rates are attributed to the introduction of PbO_2_ and the vacancy-defect graphene structure. These structural features contribute to improved reaction kinetics and optimized electron distribution, making the catalyst highly efficient for OER. The activation reaction surface area of NiFe-PbO_2_/S/rGO-45, as shown in Figure S5, is the largest, indicating that the introduction of graphene and vacancy defects increases the catalyst’s reactive surface area. This indicates that vacancy defects and the graphene matrix collectively significantly enhance the catalyst’s performance. Furthermore, following 50 h of continuous potential assessment, the current density of NiFe-PbO_2_/S/rGO-45 exhibited minimal decline (Figure 6d), demonstrating its excellent stability. This suggests that the vacancy-defect structure not only improves the catalyst’s reaction kinetics but also contributes to its long-term durability.

To further verify the exceptional activity of NiFe-PbO_2_/S/rGO-45 and explore the changes during the OER process, XRD characterization was performed on the samples before and after 50 h of potentiostatic testing (Figure S6). The results show a small amount of Fe_3_O_4_ formation after the reaction, which likely exposes iron active sites and facilitates water molecule adsorption. SEM observations (Figure S7) reveal surface degradation and nanoparticle aggregation post-OER testing, indicating structural changes after prolonged testing. ICP-MS analysis (Figure S8) of the concentrations of Ni, Fe, Pb, and S during the CV activation process reveals that Ni, Fe, and Pb dissolve and redeposit in the electrolyte, while sulfur dissolves only slightly and undergoes a gradual dissolution process, which is beneficial for the sample’s stability.

**Figure 7:**
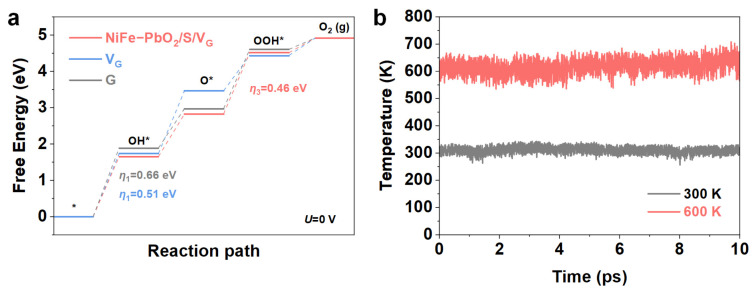
(**a**) OER free-energy diagram of NiFe-PbO_2_/S/V_G_ and V_G_, (**b**) Temperature fluctuation for NiFe-PbO_2_/S/V_G_.

Building on these experimental findings, DFT calculations were performed to assess the impact of defect structures on catalytic performance. Based on the XRD analysis, the (003) crystal plane, most exposed to NiFe-LDH, was chosen for modeling. The NiFe-PbO_2_/S/V_G_ and V_G_ heterojunction models, which include abundant defect structures (with vacancies, V, and graphene, G), were constructed for the typical OER reaction model [42]. The Gibbs free energy curve demonstrates that the introduction of vacancies in graphene reduces ΔG_OH_ and lowers overpotential. The presence of sulfur vacancies improves adsorption efficiency, while the addition of oxygen further stabilizes the structure, altering the rate-determining step of the OER from ΔG_OH_ in V_G_ to ΔG_OOH_ in NiFe-PbO_2_/S/V_G_. The overpotential of the rate-determining step for NiFe-PbO_2_/S/V_G_ is 0.46 eV (Figure 7a). Vacancies substantially influence the electrical structure and conductivity, optimizing the adsorption of OER intermediates, hence improving total activity. Vacancy structures create extra active sites, enhancing the adsorption and reactivity of intermediates, while alterations in electronic structure improve charge transfer kinetics, hence augmenting OER performance [43]. In addition, NiFe-PbO_2_/S/V_G_ exhibits good thermal stability as demonstrated by small total energy and temperature fluctuations in AIMD simulations (Figure 7b and Figure S9). Furthermore, the material maintains its structural integrity, with stable vacancy configurations, contributing to its overall robustness.

## 4. Conclusions

In summary, NiFe-PbO_2_/S/rGO-45, a newly conceived 2D material system, was manufactured via RF plasma, allowing for meticulous control over the material’s characteristics. The integration of a graphene framework improved the catalyst’s conductivity, while the plasma etching process reduced GO to rGO, facilitating the loading of NiFe LDH and PbO_2_ as well as sulfur doping. This plasma-assisted reduction also generated carbon defects and sulfur vacancies, which promote charge transfer, and enhance the distribution of active sites, collectively accelerating the surface OER process. As a result, NiFe-PbO_2_/S/rGO-45 exhibited superior OER performance compared to commercial IrO_2_, with a η_50_ of 230 mV. The novelty of this material system lies in the combination of the graphene framework and the layered structures of NiFe-LDH and PbO_2_, enhancing the catalyst’s performance. In addition, the RF plasma-assisted synthesis method is scalable and adaptable to industrial-scale manufacturing processes. The use of abundant and inexpensive transition metals, combined with the graphene framework, makes the NiFe-PbO_2_/S/rGO material system an economically viable alternative to precious metal-based catalysts. The superior OER performance demonstrated has the potential to greatly enhance the efficiency and cost-effectiveness of hydrogen production through water electrolysis, contributing to the global efforts in transitioning towards a sustainable and low-carbon energy future.

## Data Availability

Data are contained within the article or Supplementary Material.

## Supplementary Materials

The following supporting information can be downloaded at https://www.mdpi.com/article/10.3390/ma18010121/s1, Figure S1: EDS pattern of NiFe-PbO_2_/S/rGO-45. Figure S2: Particle size of NiFe-PbO_2_/S/rGO-45. Figure S3: XPS survey spectrum of NiFe-PbO_2_/S/rGO-45. Figure S4: Nyquist plot of NiFe-PbO_2_/S/rGO-X. Figure S5: Capacitive current density against scan rates of NiFe-PbO_2_/S/rGO-X. Figure S6: XRD patterns of NiFe-PbO_2_/S/rGO-45 before reaction, after OER processes in 1 M KOH for 50 h. Figure S7: SEM image of NiFe-PbO_2_/S/rGO-45 before reaction, after OER for 50 h. Figure S8: Concentrations of Ni, Fe, Pb, and S in the electrolyte of NiFe-PbO_2_/S/rGO-45 prior to the reaction and subsequent to the OER processes in 1 M KOH for 50 h, as determined by ICP-MS analysis. Figure S9: Energy fluctuation for NiFe-PbO_2_/S/V_G_ at (a) 600 K and (b) 300 K.

## References

[1] Jawhari A.H., Hasan N. (2023). Nanocomposite Electrocatalysts for Hydrogen Evolution Reactions (HERs) for Sustainable and Efficient Hydrogen Energy-Future Prospects. Materials.

[2] Popov A.A., Afonnikova S.D., Varygin A.D., Bauman Y.I., Trenikhin M.V., Plyusnin P.E., Shubin Y.V., Vedyagin A.A., Mishakov I.V. (2023). Pt1-xNix Alloy Nanoparticles Embedded in Self-Grown Carbon Nanofibers: Synthesis, Properties and Catalytic Activity in HER. Catalysts.

[3] Shamskhou K., Awada H., Yari F., Aljabour A., Schöfberger W. (2023). A Molecular Binuclear Nickel (II) Schiff Base Complex for Efficient HER Electrocatalysis. Catalysts.

[4] Seenivasan S., Seo J. (2023). Inverting destructive electrochemical reconstruction of niobium nitride catalyst to construct highly efficient HER/OER catalyst. Chem. Eng. J..

[5] Xin Y.M., Hua Q.Q., Li C.J., Zhu H.D., Gao L.G., Ren X.F., Yang P.X., Liu A.M. (2024). Enhancing electrochemical performance and corrosion resistance of nickel-based catalysts in seawater electrolysis: Focusing on OER and HER. J. Mater. Chem. A.

[6] Gan J.C., Jiang Z.F., Fang K.M., Li X.S., Zhang L., Feng J.J., Wang A.J. (2025). Low Rh doping accelerated HER/OER bifunctional catalytic activities of nanoflower-like Ni-Co sulfide for greatly boosting overall water splitting. J. Colloid Interface Sci..

[7] Khan I., Baig N., Bake A., Haroon M., Ashraf M., Al-Saadi A., Tahir M.N., Wooh S. (2023). Robust electrocatalysts decorated three-dimensional laser-induced graphene for selective alkaline OER and HER. Carbon.

[8] Liao Y.Y., He R.C., Pan W.H., Li Y., Wang Y.Y., Li J., Li Y.X. (2023). Lattice distortion induced Ce-doped NiFe-LDH for efficient oxygen evolution. Chem. Eng. J..

[9] Shi J.W., He H.W., Guo Y.H., Ji F., Li J., Zhang Y., Deng C.W., Fan L.Y., Cai W.W. (2023). Enabling high-efficiency ethanol oxidation on NiFe-LDH via deprotonation promotion and absorption inhibition. J. Energy Chem..

[10] Li M.Y., Yang J.K., Liang S., Hou H.J., Hu J.P., Liu B.C., Kumar R.V. (2019). Review on clean recovery of discarded/spent lead-acid battery and trends of recycled products. J. Power Sources.

[11] Hakimi F., Ghalkhani M., Rashchi F., Dolati A. (2024). Pulse electrodeposition synthesis of Ti/PbO_2_-IrO_2_ nano-composite electrode to restrict the OER in the zinc electrowinning. J. Environ. Chem. Eng..

[12] Lin L., Wang Y.F., Ye Q., Zhao Y.X., Cheng Y.L. (2023). Rapid fabrication of Fe_x_Ni_2−x_P_4_O_12_ and graphene hybrids as electrocatalyst for highly efficient oxygen evolution reaction. Appl. Catal. B-Environ. Energy.

[13] González-Ingelmo M., García M.L., Oropeza F.E., Alvarez P., Blanco C., Santamaría R., Rocha V.G. (2023). Ultra-high dispersion of Ni-based OER catalysts on graphene 3D networks enhances the in situ Fe^3+^ catalytic activation. J. Mater. Chem. A.

[14] Gong Z.C., Liu J.J., Yan M.M., Gong H.S., Ye G.L., Fei H.L. (2023). Highly Durable and Efficient Seawater Electrolysis Enabled by Defective Graphene-Confined Nanoreactor. ACS Nano.

[15] Goyenola C., Stafström S., Hultman L., Gueorguiev G.K. (2012). Structural Patterns Arising during Synthetic Growth of Fullerene-Like Sulfocarbide. J. Phys. Chem. C.

[16] Sfuncia G., Nicotra G., Giannazzo F., Pécz B., Gueorguiev G.K., Kakanakova-Georgieva A. (2023). 2D graphitic-like gallium nitride and other structural selectivity in confinement at the graphene/SiC interface. Crystengcomm.

[17] Yang H.C., Wang C.H., Zhang Y.J., Wang Q.B. (2019). Green synthesis of NiFe LDH/Ni foam at room temperature for highly efficient electrocatalytic oxygen evolution reaction. Sci. China-Mater..

[18] Hong X.D., Li S.L., Tang X.N., Sun Z.H., Li F. (2018). Self-supporting porous CoS_2_/rGO sulfur host prepared by bottom-up assembly for lithium-sulfur batteries. J. Alloy. Compd..

[19] Pimenta M.A., Dresselhaus G., Dresselhaus M.S., Cançado L.G., Jorio A., Saito R. (2007). Studying disorder in graphite-based systems by Raman spectroscopy. Phys. Chem. Chem. Phys..

[20] Zhang Y.Q., Liang Y.M., Zhou J.X. (2014). Recent Progress of Graphene Doping. Acta Chim. Sin..

[21] Zhu J.W., Mu S.C. (2020). Defect Engineering in the Carbon-Based Electrocatalysts: Insight into the Intrinsic Carbon Defects. Adv. Funct. Mater..

[22] Huang X.X., Shen T., Zhang T., Qiu H.L., Gu X.X., Ali Z., Hou Y.L. (2020). Efficient Oxygen Reduction Catalysts of Porous Carbon Nanostructures Decorated with Transition Metal Species. Adv. Energy Mater..

[23] Kim J.H., Choi K.J., Yoon S.G. (2005). Electrical and reliability characteristics of HfO_2_ gate dielectric treated in N_2_ and NH_3_ plasma atmosphere. Appl. Surf. Sci..

[24] Petrovic M., Jovanovic T., Rancev S., Kovac J., Velinov N., Najdanovic S., Kostic M., Bojic A. (2022). Plasma modified electrosynthesized cerium oxide catalyst for plasma and photocatalytic degradation of RB 19 dye. J. Environ. Chem. Eng..

[25] Rousseau A., Guaitella O., Gatilova L., Thevenet F., Guillard C., Röpcke J., Stancu G.D. (2005). Photocatalyst activation in a pulsed low pressure discharge. Appl. Phys. Lett..

[26] Czech T., Sobczyk A.T., Jaworek A. (2011). Optical emission spectroscopy of point-plane corona and back-corona discharges in air. Eur. Phys. J. D.

[27] Lu L.L., Xu X.X., An K.L., Wang Y., Shi F.N. (2018). Coordination Polymer Derived NiS@g-C_3_N_4_ Composite Photocatalyst for Sulfur Vacancy and Photothermal Effect Synergistic Enhanced H2 Production. ACS Sustain. Chem. Eng..

[28] Xie J.F., Zhang H., Li S., Wang R.X., Sun X., Zhou M., Zhou J.F., Lou X.W., Xie Y. (2013). Defect-Rich MoS_2_ Ultrathin Nanosheets with Additional Active Edge Sites for Enhanced Electrocatalytic Hydrogen Evolution. Adv. Mater..

[29] Li H., Tsai C., Koh A.L., Cai L.L., Contryman A.W., Fragapane A.H., Zhao J.H., Han H.S., Manoharan H.C., Abild-Pedersen F. (2016). Activating and optimizing MoS_2_ basal planes for hydrogen evolution through the formation of strained sulphur vacancies. Nat. Mater..

[30] Bo L.L., Shi W.P., Nian F., Hu Y.S., Pu L.M., Li P., Zhang Z.X., Tong J.H. (2023). Interface engineering of Co_3_S_4_@Co_3_O_4_/N, S-doped carbon core@shell nanostructures serve as an excellent bifunctional ORR/OER electrocatalyst for rechargeable Zn-air battery. Sep. Purif. Technol..

[31] Chen D.L., Gan C.L., Fan X.Q., Zhang L., Li W., Zhu M.H., Quan X. (2019). Improving the Dynamic Mechanical Properties of XNBR Using ILs/KH550-Functionalized Multilayer Graphene. Materials.

[32] Wen P., Lei R.B., Cao X., Ma Q., Zhang G.W., Guo C.X., Wang X.W., Qiu Y.J. (2023). Anchored Ni nanocrystals boosting BiVO_4_ photoanode for highly efficient water oxidation via in-situ generation of Ni@NiOOH co-catalyst. Chem. Eng. J..

[33] Chen Q., Huang J.F., Xiao T., Cao L.Y., Liu D.H., Li X.Y., Niu M.F., Xu G.T., Kajiyoshi K., Feng L.L. (2023). V-doped Ni_2_P nanoparticle grafted g-C_3_N_4_ nanosheets for enhanced photocatalytic hydrogen evolution performance under visible light. Dalton Trans..

[34] Ye X.M., Meng X.N., Han Z.Q., Qi Y.G., Li Z.M., Tian P.P., Wang W.S., Li J., Li Y.C., Zhang W.C. (2023). Designing Fe-containing polyhedral oligomeric silsesquioxane to endow superior mechanical and flame-retardant performances of polyamide 1010. Compos. Sci. Technol..

[35] Jiang Z.X., Yu T.Q., Chen J.L., Tan K.X., Deng R., Zhou A.C., Yin S.B. (2023). Regulating Competitive Adsorption on Pt Nanoparticles by Introducing Pb to Expedite Hydrogen Production via Ammonia Oxidation. ACS Appl. Nano Mater..

[36] Hamze M., Rezaei M., Tabaian S.H. (2023). Cobalt ferrite coated on Ti/Ni/PbO_x_ with enhanced electrocatalytic stability for chloride ions-contained water splitting. J. Electroanal. Chem..

[37] Jiang G.S., Chen M.L., Sun Y.Z., Wu Y.F., Pan J.Q. (2024). Highly dispersed Ir/Fe nanoparticles anchored at nitrogen-doped activated pyrolytic carbon black as a high-performance OER catalyst for lead recovery. Dalton Trans..

[38] Li S.M., Shi M., Wu C.X., Nie K.Q., Wei Z., Jiang X.P., Liu X.B., Chen H.L., Tian X.L., Wu D.X. (2024). Surface addition of Ag on PbO_2_ to enable efficient oxygen evolution reaction in pH-neutral media. Chem. Eng. J..

[39] Wang X.B., Wang J.L., Jiang W.H., Chen C., Wei J.L., Yu B.H., Chen B.M., Xu R.D., Yang L.J. (2023). MnCo_2_O_4_ decorating porous PbO2 composite with enhanced activity and durability for acidic water oxidation. Fuel.

[40] Wang X.B., Wang J.L., Tong X.N., Wu S., Wei J.L., Chen B.M., Xu R.D., Yang L.J. (2023). Constructing of Pb-Sn/a-PbO_2_/β-PbO_2_-Co_2_MnO_4_ composite electrode for enhanced oxygen evolution and zinc electrowinning. Mater. Today Phys..

[41] Wu S., Wang J.L., Wang X.B., Jiang D., Wei J.L., Tong X.N., Liu Z.W., Kong Q.X., Zong N.X., Xu R.D. (2024). Mn_3_O_4_@C micro-flakes modified Ti/TiH_2_/β-PbO_2_ anode for accelerating oxygen evolution reaction in zinc electrowinning. Mater. Res. Bull..

[42] Bajdich M., García-Mota M., Vojvodic A., Norskov J.K., Bell A.T. (2013). Theoretical Investigation of the Activity of Cobalt Oxides for the Electrochemical Oxidation of Water. J. Am. Chem. Soc..

[43] Li X.M., Zheng K.T., Zhang J.J., Li G.N., Xu C.J. (2022). Engineering Sulfur Vacancies in Spinel-Phase Co3S4 for Effective Electrocatalysis of the Oxygen Evolution Reaction. ACS Omega.

