# Supplementary material for: Graphene Supported NiFe-LDH and PbO2 Catalysts Prepared by Plasma Process for Oxygen Evolution Reaction
Source: Materials (Basel). 2024 Dec 31;18(1):121. doi: 10.3390/ma18010121 (PMC11721827; doi:10.3390/ma18010121)
Supplement: Supplementary file 1 [file materials-18-00121-s001.zip › materials-3381393-supplementary.pdf]

Electronic Supplementary Information

Graphene supported NiFe-LDH and PbO_2 catalysts prepared by plasma for oxygen evolution reaction

Tingting Yang [1], Zheng Zhang [1], Fei Tan [1], Huayu Liu [1], Xingyu Li [1], Hongqi Wang [2], Qing Yang [3, *]

[1] *State Grid Chongqing Electric Power Company Material Branch, Chongqing 401120, China*

[2] *Chongqing Jie Chuang Electric Power Technology Company Ltd., Chongqing 400031, China*

[3] *State Key Laboratory of Power Transmission Equipment Technology, School of Electrical Engineering, Chongqing University, Chongqing 400044, China*

Supporting figures

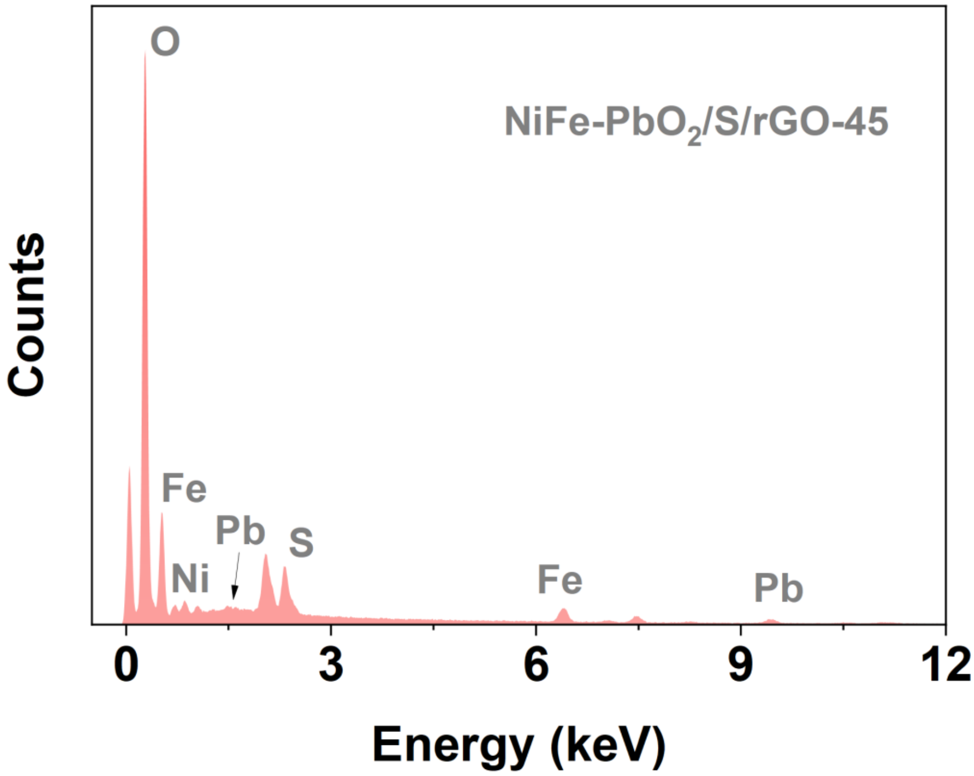

Figure S1. EDS pattern of NiFe-PbO_2/S/rGO-45.

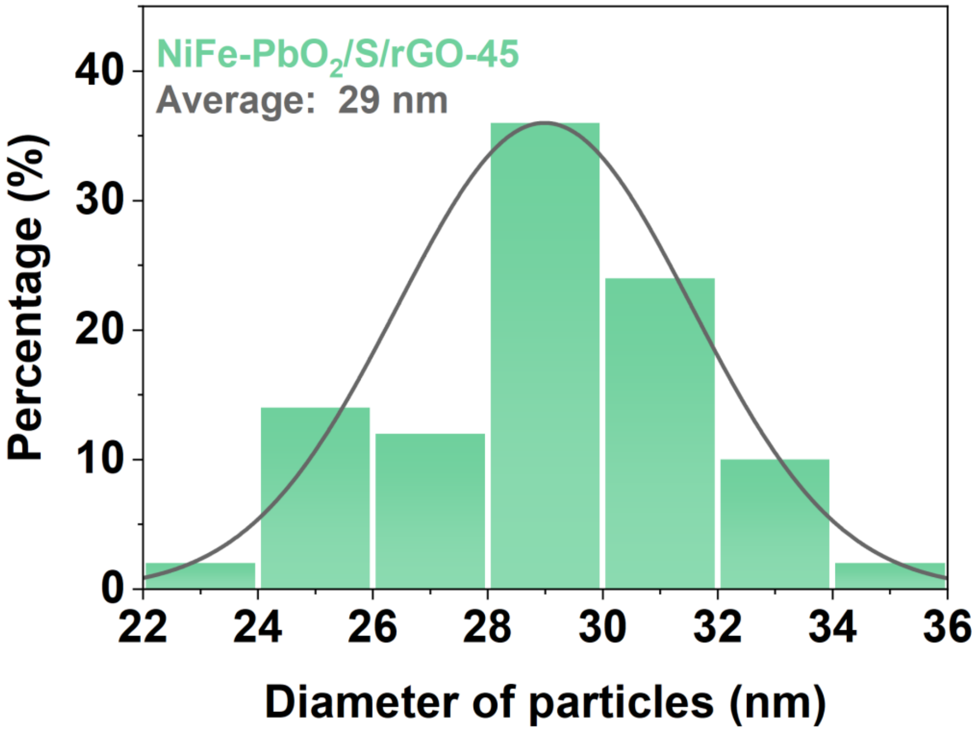

Figure S2. Particle size of NiFe-PbO_2/S/rGO-45.

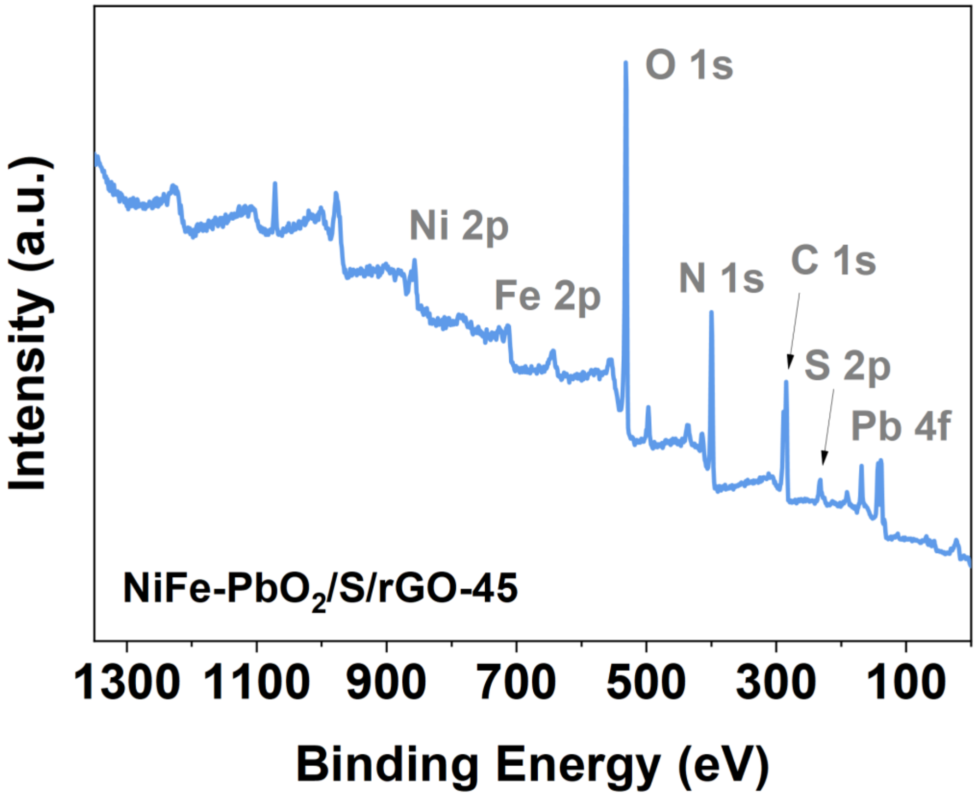

Figure S3. XPS survey spectrum of NiFe-PbO_2/S/rGO-45.

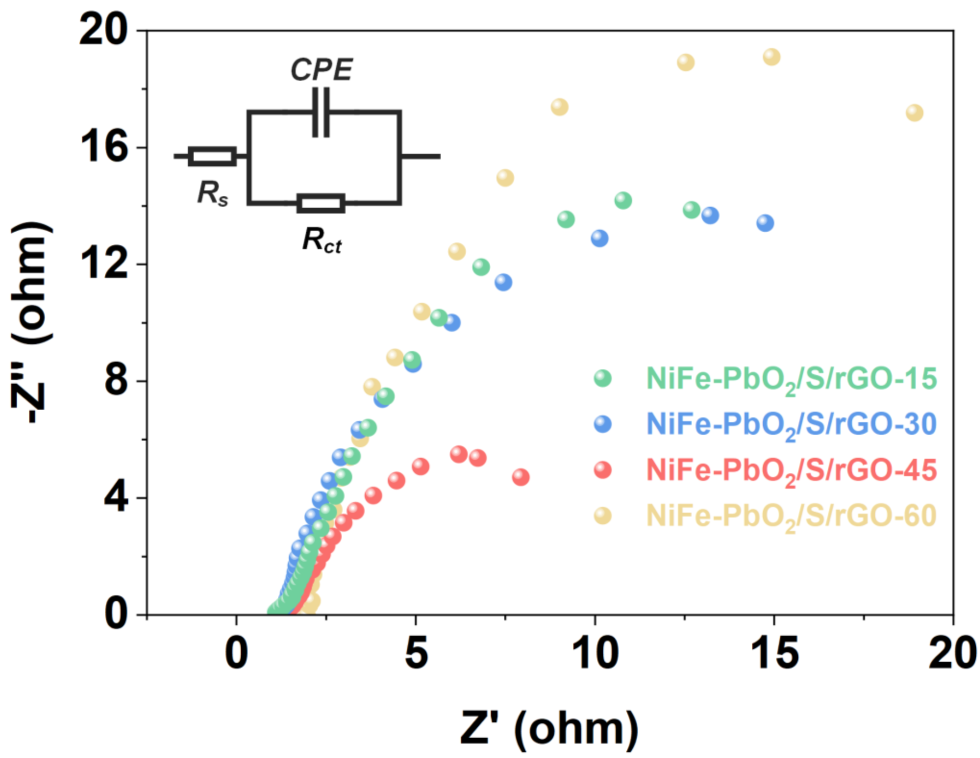

Figure S4. Nyquist plot of NiFe-PbO_2/S/rGO-X.

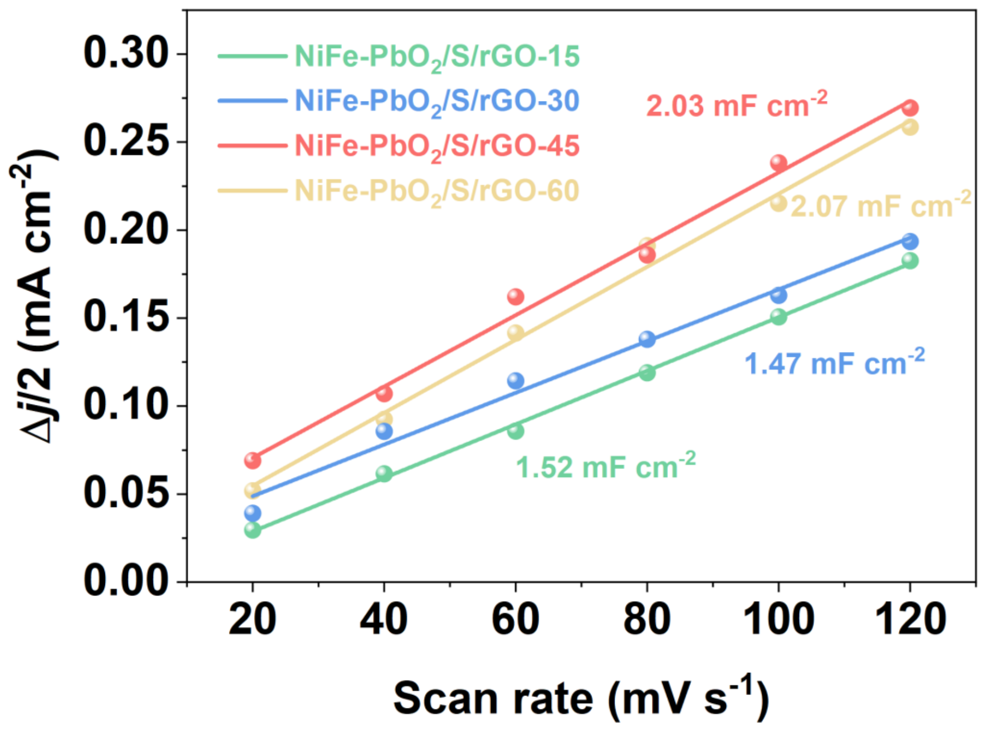

Figure S5. Capacitive current density against scan rates of NiFe-PbO_2/S/rGO-X.

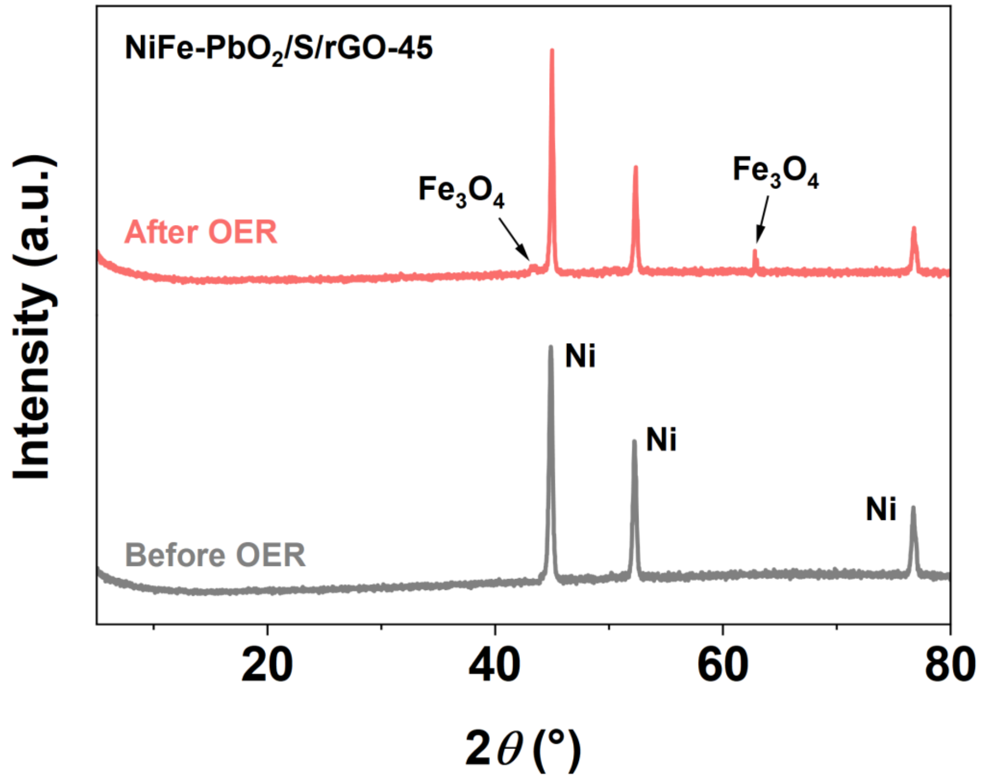

Figure S6. XRD patterns of NiFe-PbO_2/S/rGO-45 for before reaction, after OER processes in 1 M KOH for 50 h.

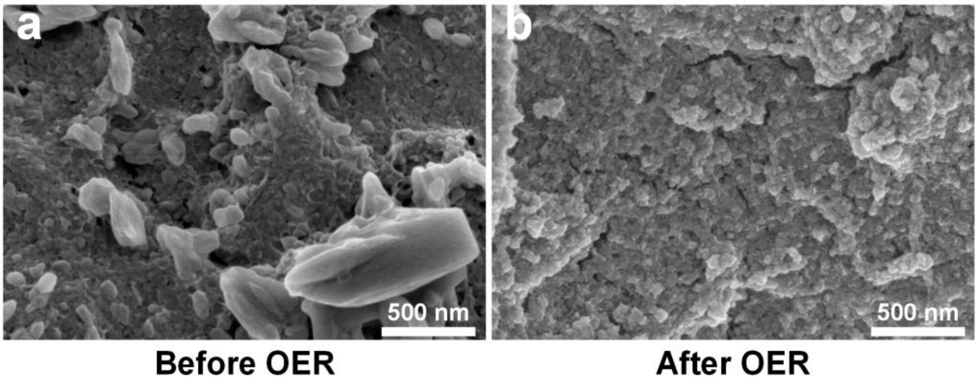

Figure S7. SEM image of NiFe-PbO_2/S/rGO-45 for before reaction, after OER processes 50 h.

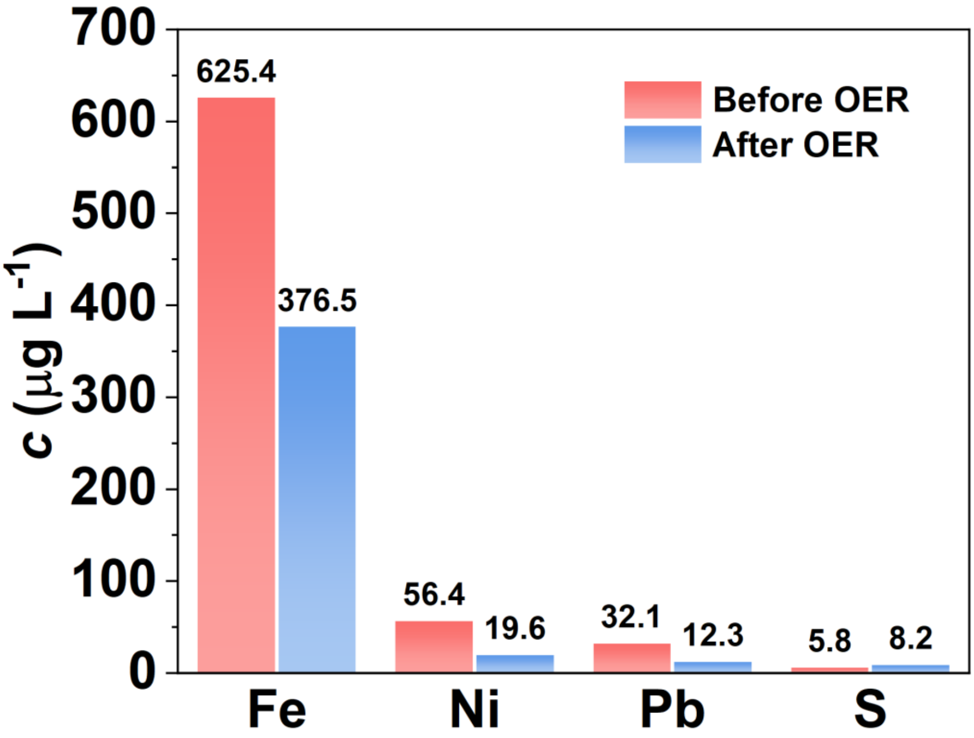

Figure S8. Ni, Fe, Pb, and S contents in the electrolyte of NiFe-PbO_2/S/rGO-45 for before reaction, after OER processes in 1 M KOH for 50 h from ICP-MS test.

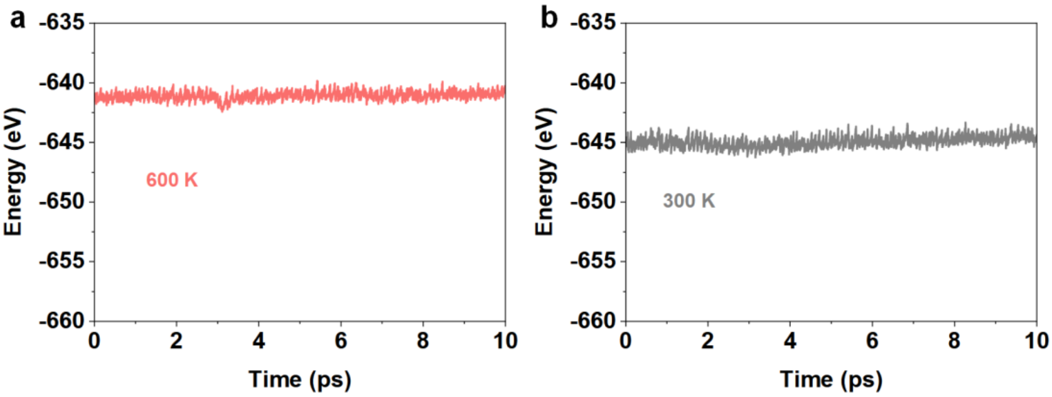

Figure S9. Energy fluctuation for NiFe-PbO_2/S/V_G at (a) 600 K and (b) 300 K.
